# Supplementary material for: A Comparative Study of Pickled Salted Eggs by Positive and Negative Pressure-Ultrasonic Method
Source: Foods. 2023 Mar 31;12(7):1477. doi: 10.3390/foods12071477 (PMC10094396; doi:10.3390/foods12071477)
Supplement: Supplementary file 1 [file foods-12-01477-s001.zip › foods-2238518-supplementary.pdf]

Supplementary material

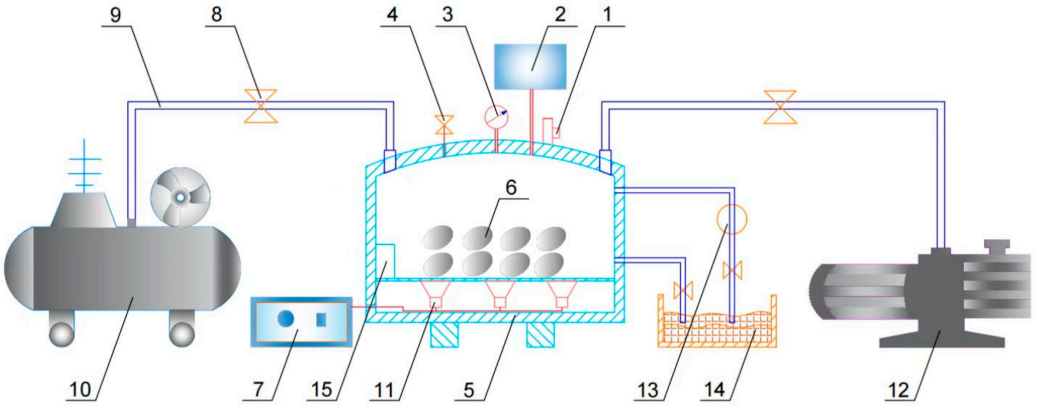

Figure S1. Rapid pickling device. 1. safety valve; 2. control cabinet; 3. pressure transmitter; 4. air valve; 5. pickled container; 6. duck egg; 7. ultrasonic generator; 8. solenoid valve; 9. pipe; 10. Compressor; 11. ultrasonic transducer; 12. vacuum machine; 13. water pump; 14. material pool; 15. temperature sensor.
